# Scatter-Hoarding Rodents Prefer Slightly Astringent Food

**DOI:** 10.1371/journal.pone.0026424

**Published:** 2011-10-26

**Authors:** Bo Wang, Jin Chen

**Affiliations:** 1 Key Laboratory of Tropical Forest Ecology, Xishuangbanna Tropical Botanical Garden, Chinese Academy of Sciences, Yunnan, China; 2 Graduate School of Chinese Academy of Sciences, Beijing, China; University of Western Ontario, Canada

## Abstract

The mutualistic interaction between scatter-hoarding rodents and their seed plants is highly complex yet poorly understood. Plants may benefit from the seed dispersal behavior of rodents, as long as seed consumption is minimized. In parallel, rodents may maximize foraging efficiency and cache high-quality resources for future consumption. Defensive compounds, such as tannins, are thought to be a major mechanism for plant control over rodent behavior. However, previous studies, using naturally occurring seeds, have not provided conclusive evidence supporting this hypothesis. Here, we test the importance of tannin concentrations on the scatter-hoarding behavior of rodents by using an artificial seed system. We combined feeding trials and field observations to examine the overall impact of seed tannin concentrations on rodent behavior and health. We found that rodents favored seeds with an intermediate amount of tannin (∼5%) in the field. Meanwhile, in rodents that were fed a diet with different tannin content, only diets with high tannin content (25%, 15%, and 10%) caused a significant negative influence on rodent survival and health. Significant differences were not found among treatments with tannin levels of 0–5%. In contrast to many existing studies, our results clearly demonstrate that scatter-hoarding rodents prefer slightly ‘astringent’ food. In the co-evolutionary arms race between plants and animals, our results suggest that while tannins may play a significant role in reducing general predation levels by the faunal community, they have no precise control over the behavior of their mutualistic partner. Instead, the two partners appear to have reached an evolutionary point where both parties receive adequate benefits, with the year-to-year outcome being dependent on a wide range of factors beyond the control of either partner.

## Introduction

Tannins are a diverse group of water-soluble phenolic compounds that have a high affinity for proteins, and are widely distributed in various parts of plants. One of the roles of tannins in plant tissues is to provide defense against herbivorous animals. Tannins have been shown to reduce the ability of animals to digest proteins [1] and/or act as potential toxins that cause direct detrimental effects to gastrointestinal mucosa and epithelia [2], in addition to kidney and liver failure [3], intrinsic nitrogen loss [4], and the disturbance of sodium balance [5], [6].

Scatter-hoarding rodents act as both predators and dispersers, and play an important role in the seed-to-seedling phase of many plant species [7]. The influence of tannin levels on the selection of seeds by rodents has attracted much attention, because such information could help determine the dynamics of plant-rodent interactions and the evolution of tannin in plant seeds. However, studies on tannin concentrations that influence rodent seed preference in the field have produced contradictory results [8]–[11]. Some studies suggest that rodents prefer to cache seeds with high tannin content and consume those with low tannin content *in situ*. One hypothesis that has been proposed to interpret this phenomenon is that high tannin in seeds may enhance their storability [8], with high tannin concentrations possibly degrading during the cached period [8], [12]. Alternatively, tannins could be correlated to the germination schedule, and rodents are selecting seeds with delayed germination rather than tannin itself [8], [13]. However, other studies have found that rodents prefer to transport seeds with low tannin content and consume seeds with high tannin concentration *in situ* [10]. Hence, in a previous study, our research group used artificial seeds to demonstrate experimentally that rodent caching behavior based on seed tannin content is inconsistent across years [11]. Consequently, we proposed that the rodent caching behavior in response to tannin content is probably a multivariate response to different environment factors [11].

To further explore how seed tannin concentrations influence rodent foraging behavior, we manipulated seed abundance and the tannin content levels of background seeds by using an artificial seed system similar to that developed in our previous study [11]. Furthermore, to better understand how tannin concentrations in food affect rodent physiology and health, we fed 2 dominant rodent species (*Apodemus latronum* and *Apodemus chevrieri*) diets containing different tannin concentrations.

## Results

### Effect of background tannin levels on rodent foraging preferences

**Figure 1:**
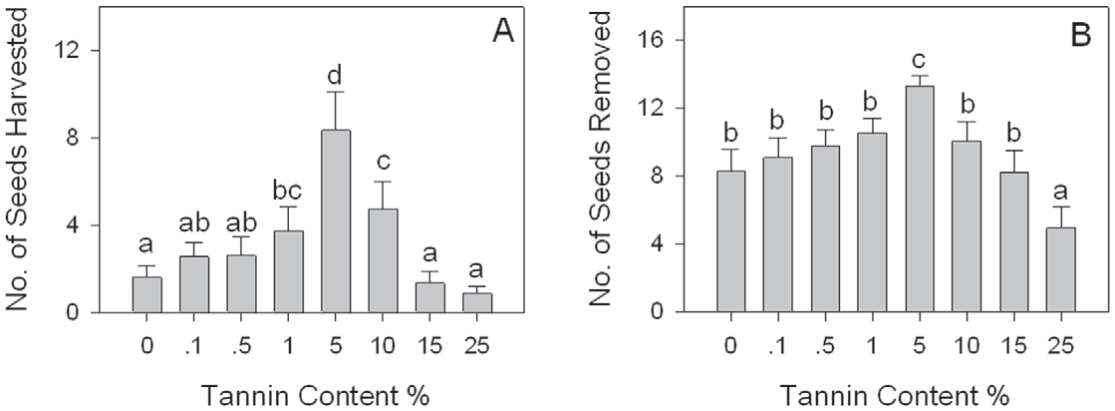
Differences of seeds harvested and removed by rodents at different tannin content backgrounds in the field (Experiment 1). Different letters indicate significant between-treatment differences (GLM Multiple Comparison, *P*<0.05). For each tannin content level the sample size was 15 (3 background levels×5 plots).

**Figure 2:**
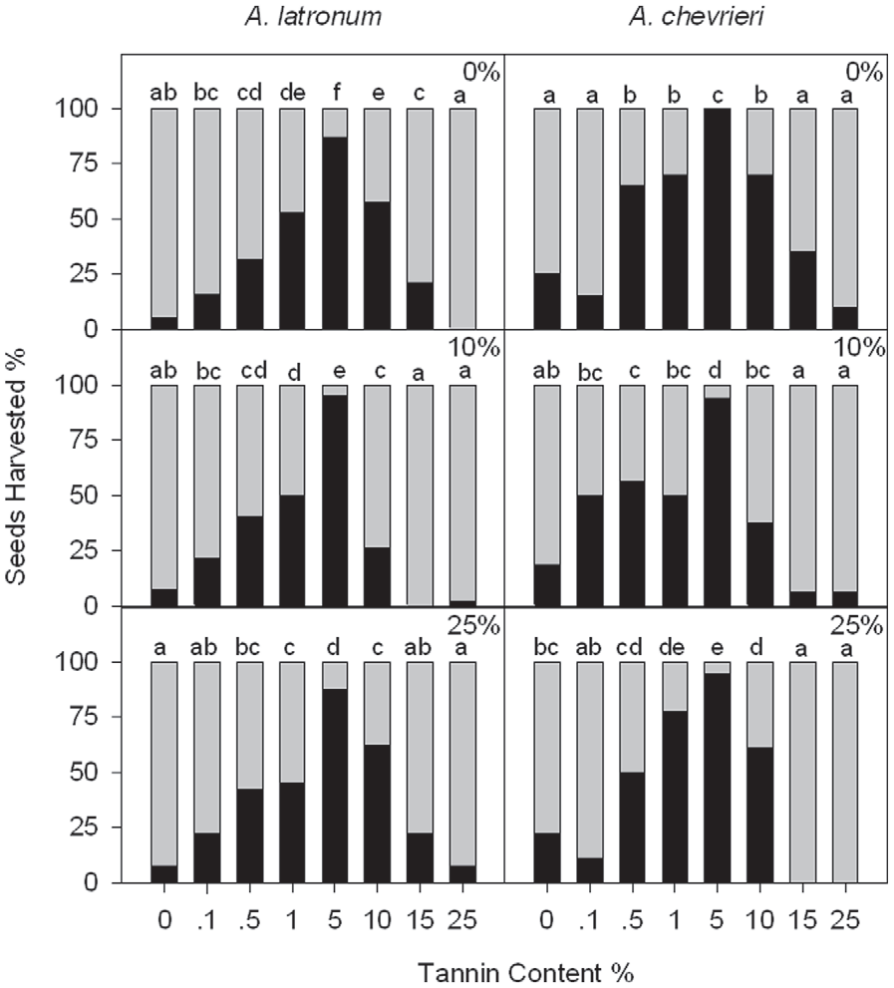
Differences of seeds harvested by rodents at different tannin content backgrounds in the enclosure (Experiment 2). Black bars represent seeds harvested by the rodents, while gray bars represent seeds that were not harvested. Different letters indicate significant between-treatment differences (pair-wise Chi-squared test, *P*<0.05). For *A. latronum* in the enclosures, the total number of seeds for each of the 8 tannin treatments was 38, 42 and 40 at tannin background levels of 0%, 10%, and 25%, respectively; while for *A. chevrieri*, the seed number was 20, 16, and 18 at tannin background levels of 0%, 10%, and 25%, respectively.

The tannin content of background seeds did not significantly influence rodent behavior in the field or in the enclosure (Experiments 1 & 2). Rodents harvested seeds with 5% tannin content much faster than seeds with lower or higher tannin content under all 3 seed background levels with tannin content in both the field (Fig. 1A, Table S1) and enclosure experiment (Fig. 2). Seeds with 5% tannin content were removed more often and eaten less *in situ* than seeds of other tannin content levels under all the 3 background levels in the field (Fig. 1B, Table S2). The dispersal distance of seeds with different tannin contents was not significantly different (*F*_7, 514_ = 1.566, *P* = 0.144), whereas the interaction among the tannin content of seeds, the background, and the plot was significant (*F* ^41, 514^  = 1.705, *P* = 0.006) (Table S3).

### Effect of seed abundance on rodent foraging preferences

**Figure 3:**
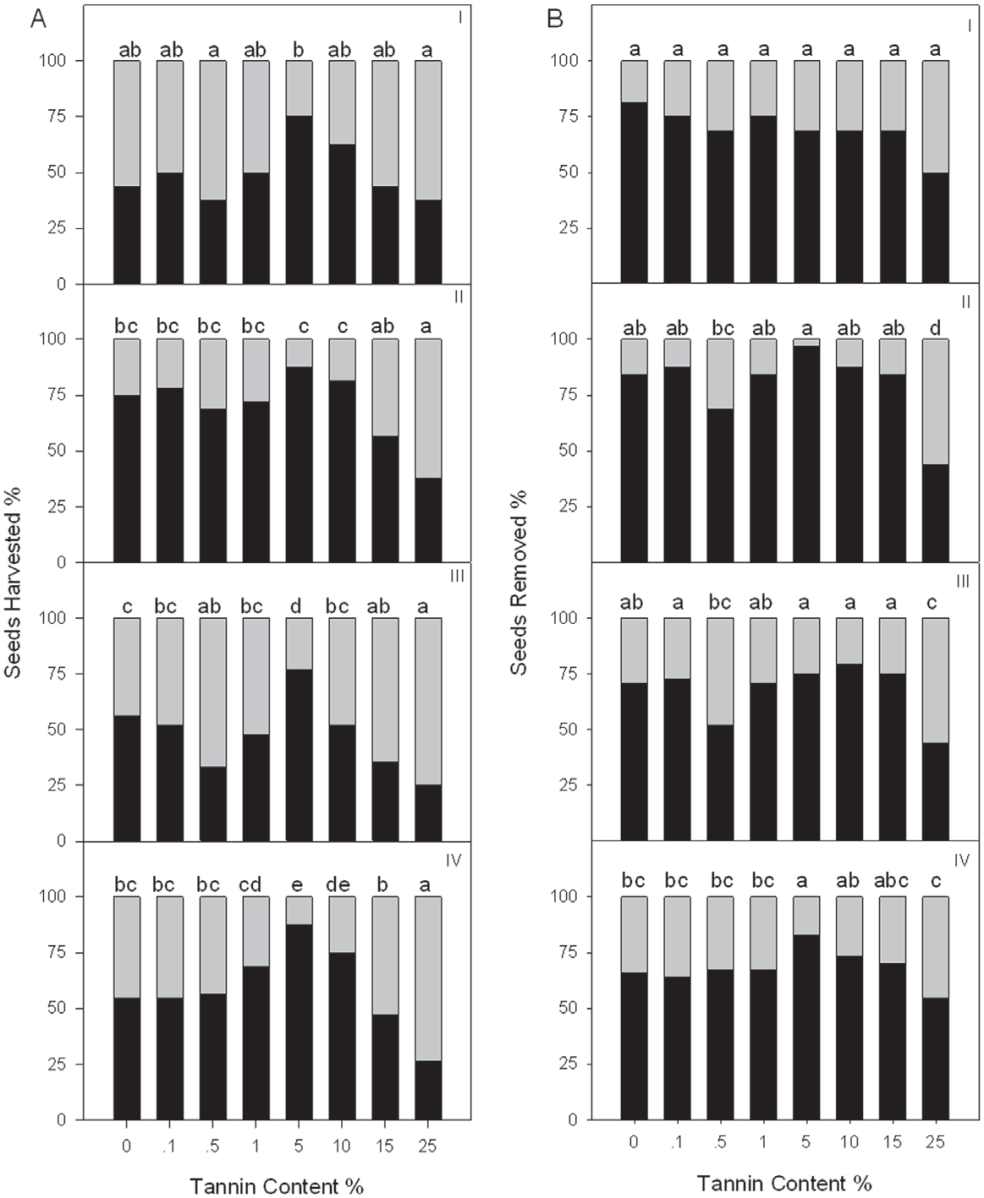
Differences of seeds harvested and removed by the rodents at different seed abundance levels in the field (Experiment 3). Four different food abundance levels were designated: I: 8 experimental seeds (1×8 tannin content levels); II: 16 seeds (2×8 tannin content levels); III: 24 seeds (3×8 tannin content levels); IV: 32 seeds (4×8 tannin content levels). In A, the black bars represent the seeds harvested by rodents, while the gray bars represent the seeds that were not harvested. In B, the black bars represent seeds removed by the rodents, while the gray bars represent seeds that were eaten. Different letters indicate significant between-treatment differences (pair-wise Chi-squared test, *P*<0.05). The total number of seeds at each tannin content level was 16, 32, 48, and 64 under each food abundance level (i.e., I, II, III and IV), respectively.

**Figure 4:**
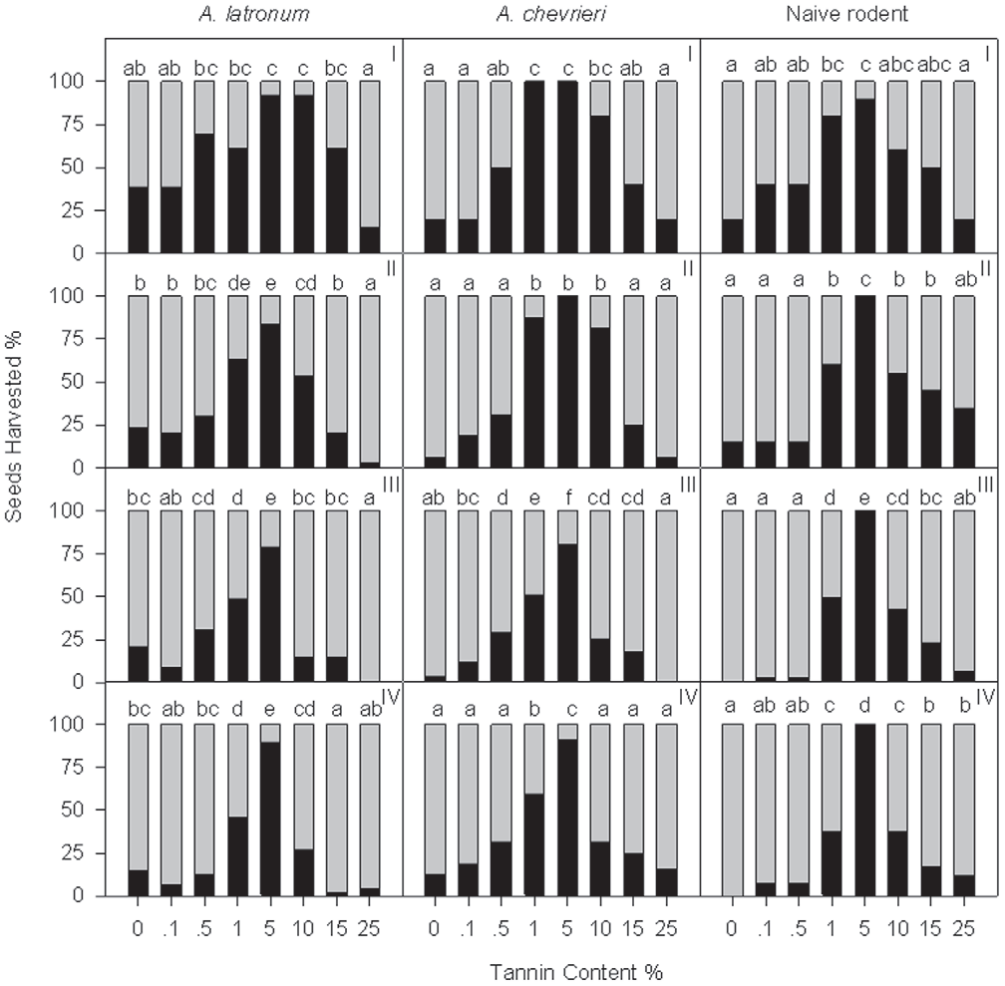
Differences of seeds harvested by the rodents at different seed abundance levels in the enclosure (Experiment 4). Black bars represent seeds harvested, while gray bars represent seeds that were not harvested. Different letters indicate significant between-treatment differences (pair-wise Chi-squared test, *P*<0.05). For *A. latronum*, the total number of seeds was 13, 30, 33 and 48; for *A. chevrieri*, the total number of seeds was 10, 16, 51 and 32; and for the non-experienced mice, the total number of seeds was 10, 20, 30 and 40, under each food abundance level (i.e., I, II, III and IV), respectively.

Seed abundance did not significantly influence the seed preferences of rodents (Experiments 3 & 4). Rodents harvested seeds of 5% tannin content much faster than other seeds at all volumes of food abundance in both the field (Fig. 3A) and enclosure experiment (Fig. 4). In addition, naive rodents also preferred the seeds of 5% tannin content (Fig. 4). The seeds of 5% tannin content were removed significantly more often and eaten less *in situ* than seeds of other tannin content levels at abundances II and IV but not at abundance I (Fig. 3B). The dispersal distance of seeds with different tannin content levels was not significantly different (*F*_7, 417_ = 0.505, *P* = 0.830) (Table S4).

### Effects of dietary tannin content on rodent survival and health

The dietary tannin content had a significant influence on rodent survival (Experiment 5). All rodents died on the 5^th^ day of being fed 25% and 15% tannin diet treatments. At the end of the experiments, 3 individuals survived each of the 0% and 5% tannin diet treatments, while just 1–2 individuals survived the remaining treatments (Table S5).

Form a pairwise Gehan-Wilcoxon test for the survival time of rodents with different tannin treatment, rodents with 25% tannin content had a significantly shorter survival time than all other treatments (*P*<0.05). Rodents with 15% tannin had a marginally significant shorter survival time than treatments with 1% and 5% tannin content (*P* = 0.088 and 0.109, respectively). Other treatments had no significant differences in survival time.

**Figure 5:**
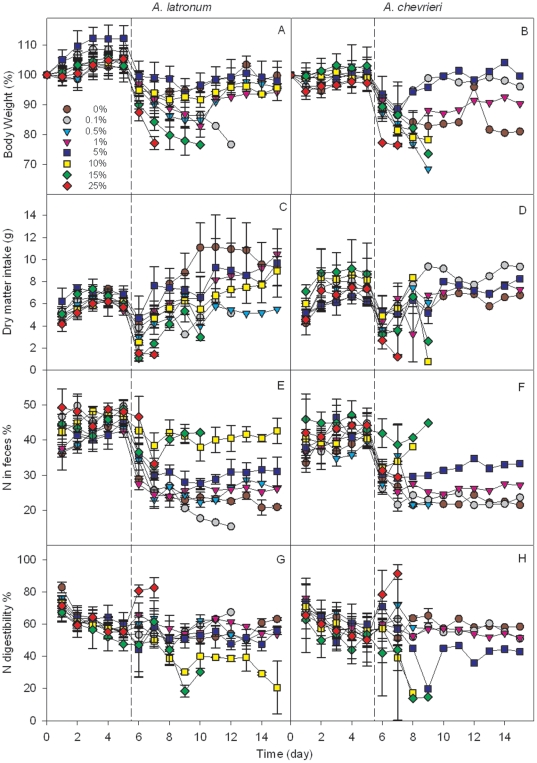
Changes in body weight and N digestibility of the experimental rodents. The bars represent mean ± SE. Body weight was expressed as a ratio to initial body weight on day 0. Laboratory chow was provided from day 0 to day 4. Experimental food with different tannin content (0%, 0.1%, 0.5%, 1%, 5%, 10%, 15% and 25%) was provided from day 5 to day 14, and the physiological indexes changed at day 6 (the dashed lines). Five rodents (3 *A. latronum* and 2 *A. chevrieri*) were used for each diet type. All *A. latronum* supplied with 0.1%, 15%, and 25% tannin content diets and all *A. chevrieri* supplied with 0.5%, 10%, 15%, and 25% tannin content diets died during the experimental period. At the end of the experiment, a total of 2, 1, 1, 2, and 2 individuals of *A. latronum* survived that were supplied with 0%, 0.5%, 1%, 5%, and 10% tannin content diets, respectively, while 1 individual of *A. chevrieri* survived in each group supplied with 0%, 0.1%, 1%, and 5%F tannin content diets.

With respect to body weight and other indicators of rodent physiology, the overall pattern was similar to that recorded for the survival rate and survival time. The performance of rodents fed diets with 15% and 10% tannin content was noticeably lower in comparison to the other treatments (Fig. 5). At the 8^th^ day, (i.e. the 3^rd^ day after the experimental diets were supplied), the body weight of rodents fed diets with 5% tannin content was significantly higher than those fed 15%, 1%, and 0.5% treatments. High tannin treatment (10% and 15%) rodents also showed significantly higher N content in the feces and lower N digestibility. There were no significant differences for dry matter intake among the different tannin treatments (Fig. S1).

## Discussion

In this study, the predominant selection of seeds with intermediate tannin content (5%) was exactly in accordance with the feeding experiment. From our previous study, we noticed that seeds with 5% tannin content were not excluded by rodents in 2006 and 2007. In 2006, seeds with lower tannin content (≤5%) were eaten *in situ* significantly more often than higher tannin content seeds (≥10%). In 2007, seeds with lower tannin content (≤5%) were dispersed farther than higher tannin content seeds (≥10%). Meanwhile, little differences were found in the preference of seeds with tannin content ranging from 0–5% [11].

In this study, the survival time of rodents fed diets with 25% tannin content was significantly shorter than all other treatments. In addition, the health of rodents fed diets with 15% and 10% tannin content was significantly negatively impacted. However, little difference was found among individuals given low tannin content treatments (0%–5%), with respect to changes in body weight, dry matter intake, N in feces, and N digestibility (Fig. 5, Fig. S1). Overall, these results demonstrate that rodents are able to tolerate certain amounts of tannin within their diets, exhibiting the same health and survival as rodents that have no tannin in their diets; however, these results conflict with most existing studies [3], [5], [6], [14]. At the end of the 15-day feeding experiment, some individuals died in all the treatments, including that of 0% tannin content. Hence, the deaths may be attributed to the experimental diets that were used. To manipulate different tannin contents, we added 0–30% fiber to retain the same amount of lab chow in each treatment. Therefore, high fiber content may also have certain negative impact on rodent health.

Some studies have suggested that tannin might be beneficial to some animals. For example, in rats polymeric grape seed tannins have been found to function as antioxidants *in vivo*, negating the effects of the oxidative stress that is induced by both vitamin E deficiency and atherogenic diets [15]. In addition, tannins are thought to prevent bats from contracting diseases of excessive absorption and storage of dietary iron, because tannin may bind to iron and block its absorption [16]. Tannins might also reduce the ingestion of iron for rats [17]. However, most studies suggest that tannins reduce the ability of animal to digest protein [6] or act as potential toxins that cause direct detrimental effects to gastrointestinal mucosa and epithelia [2], in addition to kidney and liver failure [3], intrinsic nitrogen loss [4], and the disturbance of sodium balance, or even death [5], [6].

Nevertheless, our study suggests that scatter-hoarding rodents in this alpine forest system have developed some physiological and/or behavioral mechanisms that might overcome the negative effects of tannins. In our study area, seeds with certain amounts of tannin are quite common, and among the 11 species analyzed, tannin concentrations ranged from 0 to 26.5% (mean 7.8%±2.9), of which just one species had no tannin content [18]. The mechanism for tannin tolerance may help rodents to utilize a wider range of edible resources, especially in this alpine forest where rodents are often subject to food shortages in excess of five months during the winter. Other explanations for why rodents favor intermediate tannin contents in this area require further investigation.

As discussed in our previous study [11], the seed abundance and tannin content of background seeds may influence the response of rodents to seeds of different tannin content levels. The artificial seeds that we released in the field during the experiments may be not enough to change the seed abundance and background level of tannin content in the whole area, or the whole forest where rodents live. Hence, it may be not definitive to state that neither background tannin levels nor the seed abundance affect rodent foraging behavior in relation to tannin content. However, the results from the enclosure experiments were identical to our field experiments. During the enclosure experiments, an ample supply of seeds was provided per rodent individual each night. Based on these results, we are confident that background tannin levels and seed abundance do not affect rodent foraging behavior with respect to the selection of seeds of different tannin content.

The fact that rodents can tolerate intermediate tannin content means that plants may exert a weak influence on rodent behavior. Plants exploit the natural tendency of rodents to cache seeds for future consumption, along with their tendency to forget the cache locations. Tannins in seeds may give a plant the ability to manipulate rodent foraging behavior by increasing the likelihood that a seed will be cached, and decrease the likelihood that a seed will be eventually retrieved from the cache site and eaten (see the discussion in [19]). However, based on our previous studies, when combining the effects of seed size and some other factors, the effects of tannin content on rodent behavior varied substantially from year to year [11]. Therefore, plants may not be likely to use tannin to provide a fair control over the behavior of their mutualistic partner in this co-evolutionary arms race.

## Materials and Methods

### Ethics Statement

This study was carried out in strict accordance with the Guide for the Care and Use of Laboratory Animals of China. The protocol was approved by the Administrative Panel on the Ethics of Animal Experiments of Xishuangbanna Tropical Botanical Garden, Chinese Academy of Sciences (Permit Number: XTBG2009-001). We signed a contract (No. 20090059) with the Shangri-La Alpine Botanical Garden in 2009, and the contract included the permissions to access the study site and conduct this study.

### Study site

This study was carried out in 2009 in a natural forest in the Shangri-La Alpine Botanical Garden (27°54′ N, 99°38′ E, altitude 3456 m), Yunnan province, China, where *Pinus densata* is the dominant species. The botanical garden borders the natural forest, thus the rodent community in the study site was not isolated from the natural forest.

### Study material

As in our previous study [11], we made artificial seeds with clay, peanut powder, and tannin acid (C_76_H_52_O_46_, molecular mass 1701.23). All artificial seeds were ball-shaped with a diameter of ∼15 mm. A 15-cm thin steel thread with a numbered, small, red plastic tag (2.5×0.7 cm) was connected to each seed. We created 3 types of background seeds with tannin contents: 0%, 10%, and 25%; and 8 types kinds of experimental seeds with tannin contents: 0%, 0.1%, 0.5%, 1%, 5%, 10%, 15%, and 25%.

### Experiment design

#### Experiment 1

To explore how the tannin concentration of seeds influences rodent foraging behavior under conditions with seeds of different tannin content background, 3 different background tannin levels (i.e. 0%, 10%, and 25%) were manipulated. Five plots (3×3 m)>50 m apart were selected in the forest to test each level. At each plot, we selected 16 seed release points in a 4×4 grid, with about 1 m between points. Each of the 16 points were divided into 2 groups of 8 for experimental and background seeds. All the experimental and background seed-releasing points were arranged alternately, with 16 experimental seeds (2 seeds of each of the 8 tannin levels) or 16 background seeds (of the same tannin content level) being placed at each point. The seeds at each point were arranged in a circle (diameter ∼15 cm) with the tags located outward. We checked each plot after days 1, 2, 3, 4, 6, 8, 12, 16, 20, and 24, respectively. Each plot was used 3 times to test all the 3 tannin background levels. We set 256 seeds at each plot each time, with 3,840 seeds being used in this experiment (256 seeds×5 plots×3 times), including 1,920 experimental seeds (240×8 tannin degrees) and 1,920 background seeds (640×3 tannin backgrounds). The data that was recorded included: 1) seeds eaten in situ, leaving only plastic tags and seed fragments on the ground surface of the original release plot; 2) seeds removed, which could not be found at the original release plot, including: cached (buried intact in the soil or deposited intact on the soil surface), eaten after being transported (removed by the rodents from the original release plots before being eaten), and missing (seeds that were not found within the search area, hence with an unknown fate); 3) dispersal distance, the distance between the seed we found and its original position; and 4) seeds harvested, including seeds that were both removed and eaten *in situ*.

#### Experiment 2

Two dominant rodent species (*Apodemus latronum* and *Apodemus chevrieri*) were captured from the forest, and were set free after the experiments. We built 4 sets of 10×10 m rodent-proof enclosures out of sheet iron. The enclosures walls extended 80 cm above and 40 cm below the ground. The floors of the enclosures were cement covered with 7–8 cm of soil. All of the enclosures were covered by velaria to prevent access by birds and other animals. In each enclosure, a box (45×30×25 cm) mounted on top of a 50 cm pipe was placed in the corner as a rodent nest, and a plastic plate (diameter 12 cm) was placed near the nest as a water dish. We assigned each rodent to an enclosure, and allowed it to acclimate for 1 day. Then the 3 tannin background levels were tested for the following 3 days. The order of the 3 tannin backgrounds was rotated to ensure that each background level had an equal chance in the order. We released 16 experimental seeds (2×8 tannin levels) and 16 background seeds (with same tannin content) in the middle of each enclosure in a circle (diameter ∼15 cm), with the tags located outward at 18:00 pm, and recorded the seeds harvested at 7:00 the next morning. In this experiment, we used 22 individuals of *A. latronum* and 10 individuals of *A. chevrieri* to test the effect of tannin background on rodent seed selection. Some individuals escaped during the experiment; hence, in total, just 19, 21 and 20 individuals of *A. latronum* and 10, 8 and 9 individuals of *A. chevrieri* finished the 0%, 10%, and 25% tannin background experiments, respectively.

#### Experiment 3

To explore how seed tannin concentration influences rodent foraging behavior under different seed abundances, 4 different amounts of food abundance were manipulated: I: 8 experimental seeds (1×8 tannin levels); II: 16 seeds (2×8 tannin levels); III: 24 seeds (3×8 tannin levels); IV: 32 seeds (4×8 tannin levels). Sixteen plots (0.2×0.2 m)>25 m apart were selected in the forest to test each abundance level. We used 1,280 seeds (160×8 tannin levels) in this experiment. Seeds at each plot were arranged in a circle (diameter ∼15 cm) with the tags located outward. We checked the plots following the same method as in Experiment 1.

#### Experiment 4

We used 15 individuals of *A. latronum* and 17 individuals of *A. chevrieri* to test the effect of food abundance on rodent selection in the 4 enclosures, following the same protocol as in Experiment 2. A few individuals escaped during the experiment; hence, a total of 13, 15, 11, and 12 individuals of *A. latronum* and 10, 8, 17, and 8 individuals of *A. chevrieri* finished the I, II, III, and IV levels of seed abundance experiments, respectively. In this experiment, we also used 10 naive rodents (8 individuals of *A. latronum* and 2 individuals of *A. chevrieri*). These rodents were born from pregnant rodents caught from the forest; they were raised in captivity, and fed with no-tannin content food for about 3 months before the experiment. The four levels of food abundance were the same as that stated in Experiment 2, and the experimental set was the same as in Experiment 3.

#### Experiment 5

Twenty-four individuals of *A. latronum* and 16 individuals of *A. chevrieri* were caught from the forest, housed in separate cages (60×45×20 cm), and fed with laboratory chow (protein: 19.2%; fat: 3.1%; starch: 44.5%; fiber: 2.4%; and tannin 0%) for at least 1 month before the experiment. We used laboratory chow, tannic acid, and wood fiber to prepare 8 types of experimental diets of different tannin content: 0%, 0.1%, 0.5%, 1%, 5%, 10%, 15%, and 25%. All the materials were milled until being passed through a 1 mm screen. Each type of diet contained the same proportion (70%) of laboratory chow, with the other 30% containing fiber and tannic acid. The rodents were divided into 8 groups, with each group containing 3 individuals of *A. latronum* and 2 individuals of *A. chevrier*. Each group was fed 1 concentration level of the tannin diets. The rodents were weighed on the first day (day 0) of the experiment. They were then fed zero-tannin content laboratory chow for 5 days (day 0–4) followed by 10 consecutive days on the experimental diet. At ∼8:00 each morning, the rodents were weighed and supplied with new adequate diets and bedding paper. At this time, all remaining diets and feces were collected, dried at 70 °C, and weighed to determine food intake. Dried feces were milled, and nitrogen content was measured. Nitrogen digestibility was calculated from dry matter intake, dry feces, and the nitrogen content of feces.

### Statistical analyses

In Experiment 1, a General Linear Model (GLM) was used to analyze the number of seeds that were harvested and removed, in addition to the removal distance data, with tannin content of seeds, tannin background level, and plot as 3 fixed factors. As almost all experimental seeds were harvested by the end, we only used the data of seeds harvested on the first day after they were released to evaluate the harvest preferences of rodents with respect to seeds of different tannin content.

In Experiment 2 & 4 (i.e. the enclosure experiments), where only the harvest data was yielded, we used the counting process notation to record the number of harvested seeds for each rodent individual, and we performed a pair-wise Chi-squared test process to test the difference in the proportion of harvested seeds between each 2 of the 8 tannin content levels. During the statistical analyses, we combined all the seed harvest data of the same treatment level together.

In Experiment 3, we applied the same GLM to analyze the seed removal distance data, using distance as a dependent variable, and the tannin content of the seeds, seed abundance, and plot as 3 fixed factors. However, for the seed harvest and removal data, the sample sizes within plots were too small for plot-level analysis. Thus, we used the counting process notation to record the succession of events (i.e., whether individual seeds were harvested or not, and removed or eaten *in situ*). During the statistical analyses, we combined the data of all the 16 plots. For each seed fate process, we analyzed the explanatory effects of seed tannin content under each seed abundance level. A pair-wise Chi-squared test was used to test the difference in the proportion of seeds that were harvested or not, and removed or eaten *in situ*, between each 2 of the 8 seed tannin content levels.

In Experiment 5 (i.e., the feeding experiment), we combined all individuals of both rodent species together to perform the statistics; thus, we obtained 5 individuals for each of the 8 groups (i.e., the sample size was 5 at the group level). A Gehan-Wilcoxon test was used to test difference in survival among the different tannin content diet groups. A one-way ANOVA was used to test the differences in rodent body weight, dry matter intake, N in feces, and N digestibility among different tannin content diet groups on the 8^th^ day only (i.e., the third day after the experimental diets were supplied). The reasons for selecting the 8^th^-day data for analysis were: 1) differences should be much more distinct in the latter days because of the accumulative effects of the tannin diet; 2) many individuals died from the 9^th^ day onwards, resulting in the remaining sample size being too small to perform the analysis. All of the analyses were conducted in SPSS 13.0 for windows.

## Supporting Information

Figure S1 **Effects of different tannin content diets on the body weight and N digestibility of rodents on the 8^th^ day only (i.e., the third day after the experimental diets were supplied) in Experimental 5.**
(TIF)

Table S1 **Effects of tannin content level, background tannin level, and plot on seeds harvested by rodents in Experiment 1.**
(DOC)

Table S2 **Effects of tannin content level, background tannin level, and plot on seeds removed by rodents in Experiment 1.**
(DOC)

Table S3 **Effects of tannin content level, background tannin level, and plot on the distance of seeds transported by rodents in Experiment 1.**
(DOC)

Table S4 **Effects of tannin content level, seed abundance level, and plot on the distance of seeds transported by rodents in Experiment 3.**
(DOC)

Table S5 **Survival rate of the 5 individuals (3 of** ***A. latronum*** **and 2 of** ***A. chevrieri)*** **for each treatment in Experiment 5.**
(DOC)
