# Supplementary figures and images for: Scatter-Hoarding Rodents Prefer Slightly Astringent Food
Source: PLoS One. 2011 Oct 26;6(10):e26424. doi: 10.1371/journal.pone.0026424 (PMC3202532; doi:10.1371/journal.pone.0026424)

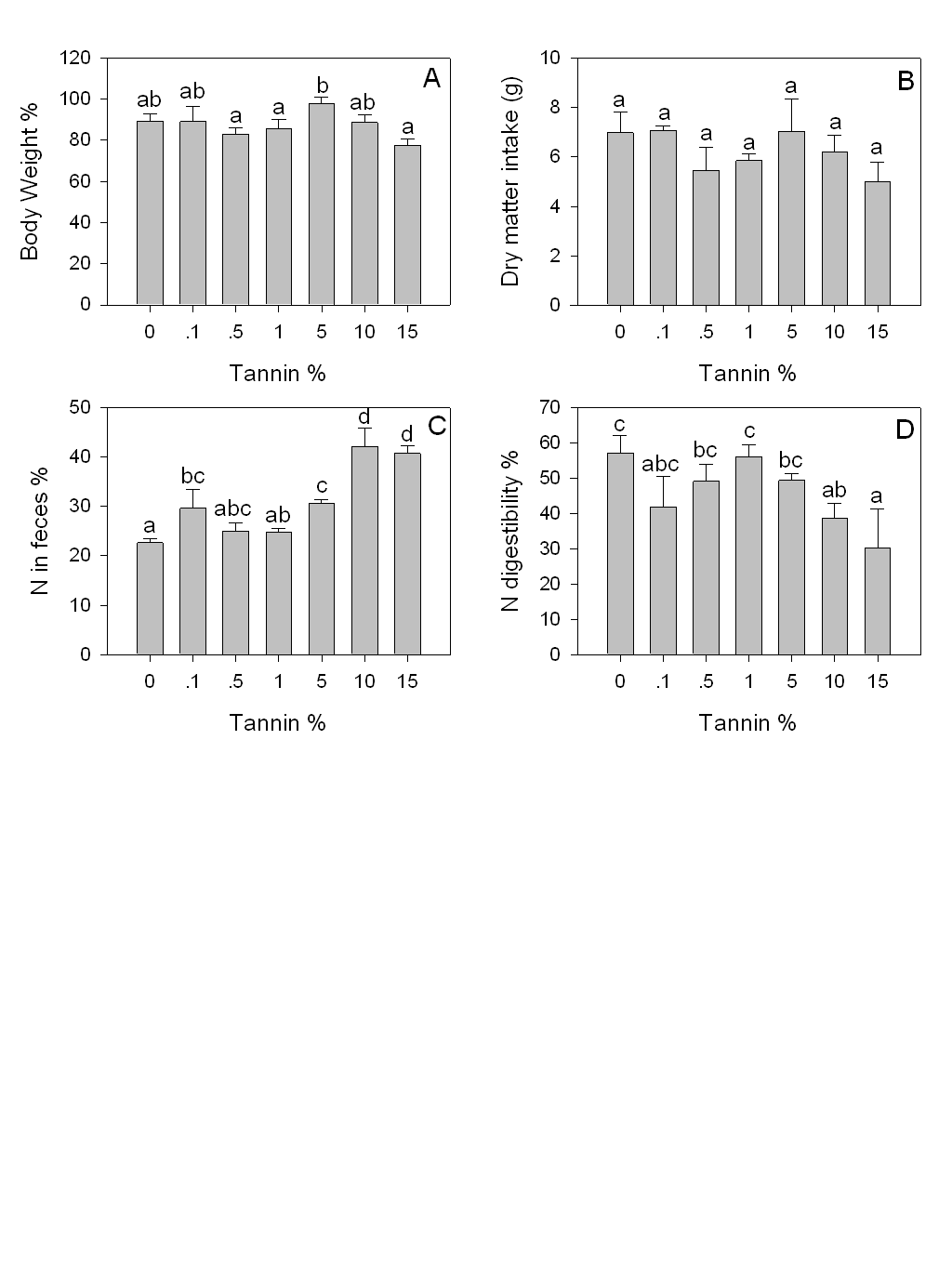

Supplement: Figure S1 — Effects of different tannin content diets on the body weight and N digestibility of rodents on the 8th day only (i.e., the third day after the experimental diets were supplied) in Experimental 5. (TIF) [file pone.0026424.s001.tif]
