# Supplementary material for: Scatter-Hoarding Rodents Prefer Slightly Astringent Food
Source: PLoS One. 2011 Oct 26;6(10):e26424. doi: 10.1371/journal.pone.0026424 (PMC3202532; doi:10.1371/journal.pone.0026424)
Supplement: Table S1 — Effects of tannin content level, background tannin level, and plot on seeds harvested by rodents in Experiment 1. (DOC) [file pone.0026424.s002.doc]

**Table S1 Effects of tannin content level, background tannin level, and plot on seeds harvested by rodents in Experiment 1.** Analyses were performed using a General Linear Model (GLM). The degrees of freedom (df), means square (MS), *F*-value (*F*), and statistical significance level (*P*) of each effect and their interaction are presented.

|  | df | MS | *F* | *P* |
| --- | --- | --- | --- | --- |
| Tannin | 7 | 88.452 | 11.127 | .000 |
| Background | 2 | 26.458 | 3.328 | .043 |
| Plot | 4 | 56.383 | 7.093 | .000 |
| Tannin * Background | 14 | 3.192 | 0.402 | .969 |
| Tannin * Plot | 28 | 7.321 | 0.921 | .584 |
| Background * Plot | 8 | 83.490 | 10.503 | .000 |
| Error | 56 | 7.949 |  |  |
| Total | 120 |  |  |  |
| Corrected Total | 119 |  |  |  |
