# Supplementary material for: Scatter-Hoarding Rodents Prefer Slightly Astringent Food
Source: PLoS One. 2011 Oct 26;6(10):e26424. doi: 10.1371/journal.pone.0026424 (PMC3202532; doi:10.1371/journal.pone.0026424)
Supplement: Table S2 — Effects of tannin content level, background tannin level, and plot on seeds removed by rodents in Experiment 1. (DOC) [file pone.0026424.s003.doc]

**Table S2 Effects of tannin content level, background tannin level, and plot on seeds removed by rodents in Experiment 1.** Analyses were performed using a General Linear Model (GLM). The degrees of freedom (df), means square (MS), *F*-value (*F*), and statistical significance level (*P*) of each effect and their interaction are presented.

|  | df | MS | *F* | *P* |
| --- | --- | --- | --- | --- |
| Tannin | 7 | 84.610 | 6.843 | .000 |
| Background | 2 | 31.033 | 2.510 | .090 |
| Plot | 4 | 62.075 | 5.021 | .002 |
| Tannin * Background | 14 | 7.252 | .587 | .864 |
| Tannin * Plot | 28 | 9.842 | .796 | .741 |
| Background * Plot | 8 | 78.669 | 6.363 | .000 |
| Error | 56 | 12.364 |  |  |
| Total | 120 |  |  |  |
| Corrected Total | 119 |  |  |  |
