# Supplementary material for: Scatter-Hoarding Rodents Prefer Slightly Astringent Food
Source: PLoS One. 2011 Oct 26;6(10):e26424. doi: 10.1371/journal.pone.0026424 (PMC3202532; doi:10.1371/journal.pone.0026424)
Supplement: Table S3 — Effects of tannin content level, background tannin level, and plot on the distance of seeds transported by rodents in Experiment 1. (DOC) [file pone.0026424.s004.doc]

**Table S3 Effects of tannin content level, background tannin level and plot on the distance of seeds transported by rodents in Experiment 1.** Analyses were performed using a General Linear Model (GLM). The degrees of freedom (df), means square (MS), *F*-value (*F*) and statistical significance level (*P*) of each effect and their interaction are presented.

|  | df | MS | *F* | *P* |
| --- | --- | --- | --- | --- |
| Tannin | 7 | 60.050 | 1.566 | .144 |
| Background | 2 | 288.137 | 7.516 | .001 |
| Plot | 4 | 292.053 | 7.618 | .000 |
| Tannin * Background | 14 | 47.760 | 1.246 | .239 |
| Tannin * Plot | 27 | 55.309 | 1.443 | .073 |
| Background * Plot | 8 | 110.619 | 2.886 | .004 |
| Tannin * Background * Plot | 41 | 65.351 | 1.705 | .006 |
| Error | 411 | 38.336 |  |  |
| Total | 515 |  |  |  |
| Corrected Total | 514 |  |  |  |
