# Supplementary material for: Scatter-Hoarding Rodents Prefer Slightly Astringent Food
Source: PLoS One. 2011 Oct 26;6(10):e26424. doi: 10.1371/journal.pone.0026424 (PMC3202532; doi:10.1371/journal.pone.0026424)
Supplement: Table S4 — Effects of tannin content level, seed abundance level, and plot on the distance of seeds transported by rodents in Experiment 3. (DOC) [file pone.0026424.s005.doc]

**Table S4 Effects of tannin content level, seed abundance level, and plot on the distance of seeds transported by rodents in Experiment 3.** Analyses were performed using a General Linear Model (GLM). The degrees of freedom (df), means square (MS), *F*-value (*F*) and statistical significance level (*P*) of each effect and their interaction are presented.

|  | df | MS | *F* | *P* |
| --- | --- | --- | --- | --- |
| Tannin | 7 | 13.833 | .505 | .830 |
| Abundance | 3 | 8.531 | .311 | .817 |
| Plot | 15 | 87.469 | 3.192 | .000 |
| Tannin * Abundance | 21 | 12.190 | .445 | .983 |
| Tannin * Plot | 96 | 16.564 | .604 | .996 |
| Abundance * Plot | 42 | 56.469 | 2.061 | .001 |
| Tannin * Abundance * Plot | 88 | 22.529 | .822 | .841 |
| Error | 145 | 27.402 |  |  |
| Total | 418 |  |  |  |
| Corrected Total | 417 |  |  |  |
