# Supplementary material for: Scatter-Hoarding Rodents Prefer Slightly Astringent Food
Source: PLoS One. 2011 Oct 26;6(10):e26424. doi: 10.1371/journal.pone.0026424 (PMC3202532; doi:10.1371/journal.pone.0026424)
Supplement: Table S5 — Survival rate of the 5 individuals (3 of A. latronum and 2 of A. chevrieri) for each treatment in Experiment 5. (DOC) [file pone.0026424.s006.doc]

**Table S5 Survival rate of the 5 individuals (3 of *A. latronum* and 2 of *A. chevrieri)* for each treatment in Experiment 5.** The rodents were fed zero-tannin content laboratory chow for 5 days (day 0–4) followed by 10 consecutive days on the experimental diet.

| Tannin % | survival individuals | | | | | | | | | | | | | | |
| --- | --- | --- | --- | --- | --- | --- | --- | --- | --- | --- | --- | --- | --- | --- | --- |
|  | day1 | day2 | day3 | day4 | day5 | day6 | day7 | day8 | day9 | day10 | day11 | day12 | day13 | day14 | day15 |
| 0 | 5 | 5 | 5 | 5 | 5 | 5 | 4 | 4 | 3 | 3 | 3 | 3 | 3 | 3 | 3 |
| 0.1 | 5 | 5 | 5 | 5 | 5 | 5 | 4 | 3 | 2 | 2 | 2 | 1 | 1 | 1 | 1 |
| 0.5 | 5 | 5 | 5 | 5 | 5 | 5 | 3 | 3 | 2 | 1 | 1 | 1 | 1 | 1 | 1 |
| 1 | 5 | 5 | 5 | 5 | 5 | 5 | 5 | 4 | 4 | 2 | 2 | 2 | 2 | 2 | 2 |
| 5 | 5 | 5 | 5 | 5 | 5 | 5 | 4 | 4 | 4 | 3 | 3 | 3 | 3 | 3 | 3 |
| 10 | 5 | 5 | 5 | 5 | 5 | 5 | 4 | 4 | 3 | 2 | 2 | 2 | 2 | 2 | 2 |
| 15 | 5 | 5 | 5 | 5 | 5 | 5 | 4 | 3 | 1 | 0 | 0 | 0 | 0 | 0 | 0 |
| 25 | 5 | 5 | 5 | 5 | 5 | 5 | 0 | 0 | 0 | 0 | 0 | 0 | 0 | 0 | 0 |
